# Exploring the experiences of struggling undergraduate medical students with formal mentoring program at a private medical college in Rawalpindi

**DOI:** 10.12669/pjms.39.3.7114

**Published:** 2023

**Authors:** Anbreen Aziz, Wajiha Shadab, Lubna Siddique, Usman Mahboob

**Affiliations:** 1 Dr. Anbreen Aziz, BDS, MHPE, AFHEA Assistant Professor, Department of Dental Education, Armed Forces Institute of Dentistry, National University of Medical Sciences (NUMS), Rawalpindi, Pakistan; 2 Dr. Wajiha Shadab, MBBS, FCPS, MHPE Associate Professor, Department of Obs/Gynae Islamic International Medical College Trust, Riphah International University, Rawalpindi, Pakistan; 3 Dr. Lubna Siddique, MBBS, MPhil Assistant Professor, Department of Physiology, Rawal Institute of Health Sciences, Islamabad, Pakistan; 4 Dr. Usman Mahboob, MBBS, MPH, FHEA, DHPE, Fellow FAIMER, Associate Professor, Institute of Health professions Education and Research (IHPER) Khyber Medical University (KMU), Peshawar, Pakistan

**Keywords:** Mentoring, Mentors, Students, Medical, Undergraduate

## Abstract

**Objective:**

To explore the experiences of struggling undergraduate medical students (mentees) with formal mentoring program at a private medical college in Rawalpindi.

**Methods:**

A qualitative exploratory study was carried out from March to August 2019. Data was collected from a purposive sample of sixteen struggling undergraduate students. Validated interview guide was utilized to conduct semi-structured one-to-one interviews. Interviews were audio recorded and transcribed accurately. Confidentiality and anonymity were ensured to the participants due to the sensitive nature of the data. Various measures were taken to achieve trustworthiness in the study. Manual thematic analysis was performed and consensus among all authors was built regarding themes and subthemes.

**Results:**

Four themes and twelve subthemes emerged from the data. Participants were satisfied with the psychosocial outcomes of the mentoring program such as emotional, moral, and psychological support, and personal and professional development. Mentees told that mentors were their best guides who shared their life experiences. Moreover, mentors provided guidance on Islam, research methods, and case-based learning. Further, mentees said that mentors provide solutions to their problems. Useful suggestions were provided by the mentees regarding betterment in the present mentoring program such as recruitment of committed staff, the need for verbal feedback from mentees about their mentors, need for career counselling and one-to-one mentoring sessions.

**Conclusions:**

Majority of the mentees were satisfied with the formal mentoring program. Mentoring focuses on personal and professional development of all medical students. In addition to the useful suggestions provided by the mentees, there is a need for the addition of specific strategies to deal with students struggling with personal or professional problems.

## INTRODUCTION

Few challenges faced by students on their entry to medical schools are: stress due to complex medical curricula, emotional immaturity and problems in adapting new surroundings.^1^ Stress is more in medical students as compared to other professions.^2^ Mentoring serves the purpose of shaping the professional and personal development of medical students.^3^ A mentor is an active partner in an ongoing mentoring relationship who helps in maximizing the mentee’s potential in reaching his personal and professional goals.^4^ In the medical context, in the absence of a formal mentoring program, students form mentoring relationships during clinical clerkships and research rotations.^5^

There is extensive variation in medical education literature about the goals of mentoring and the roles of a mentor.^6^ But there is a lack of assessment of mentoring programs hindering the detection of problems of mentors and mentees which are necessary to manage timely and appropriate support.^4^ Moreover, the assessments should be longitudinal, context-specific and goal sensitive.^4^ Formal mentoring programs are being structured without the need analysis at the institutional level. Hence, these programs are the same for all medical students without incorporation of specific strategies and approaches to solve problems of struggling medical students. Struggling medical students are those who face personal or academic problems during their training.^7^ In a previous study,^8^ perceptions of all mentees were taken regarding their experience with the mentoring program which could have masked the findings of the struggling students. The purpose of this study was to explore the experience of struggling undergraduate medical students and to strengthen mentoring programs by adding specific interventions for struggling students. Moreover, the study may have a positive impact on other medical institutes to start such programs for students’ benefit.

### Conceptual framework for mentoring:

**Fig-1:**
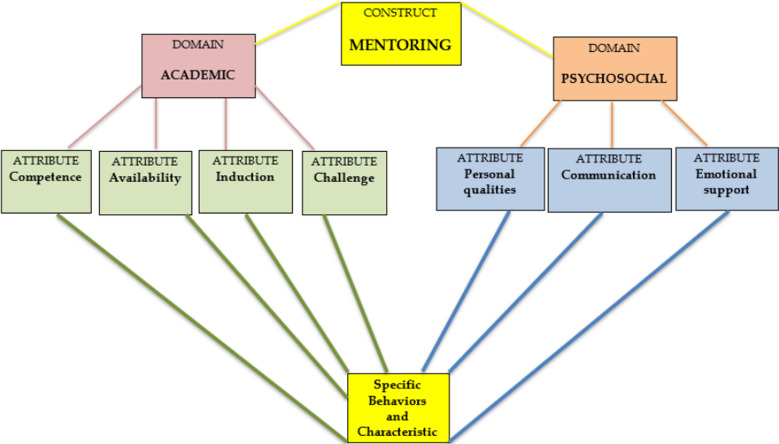
Conceptual framework of mentoring.

The conceptual framework of mentoring explained by Crawford (2012) is given in Fig.1.^9^ Academic and Psychosocial attributes of mentoring are equally important for the development of students.^10^

## METHODS

A qualitative exploratory study was carried out at a private medical college in Rawalpindi from March-August 2019. The study took place at a specific study site within a set time frame. This study focused on a mentoring phenomenon and its impact on struggling mentees in a specific setting. Ethical approval was taken from Ethics Review Committee before data collection (Riphah/IIMC/ERC/18/0313, Dated: February 11, 2019).

### Participants:

The study participants were struggling undergraduate medical students who were facing personal or academic problems. A purposive sampling technique was used to get in-depth data about the mentee’s experiences with a mentoring program and the sampling strategy is homogenous i.e., selecting a group e.g., all struggling mentees.^11^ In charge mentoring program of the institute helped in the identification of struggling mentees. Only willing struggling mentees were included in the study. The mentees who were not struggling and those who were struggling but were not willing to take part in the study were excluded.

### Data collection:

Data were collected using an interview guide which had open-ended validated questions regarding the experience of struggling mentees with the formal mentoring program, informed consent form along with interview protocols. The interview questions were piloted with three struggling students. The guide was finalized, and the data were collected through one-to-one interviews within the context to reduce recall bias. All interviews were audio recorded and transcribed accurately. Confidentiality and anonymity were ensured before the interviews. The transcripts were then anonymized before sharing with other authors for data analysis.

### Data Analysis:

Manual thematic analysis was employed because the data was less than 500 pages^11^ and patterns within the data were examined to generate themes. Initially the authors made in vivo analytic codes. Subthemes were then formed by arranging codes and consensus regarding themes and subthemes was built among all authors.

## RESULTS

Participants were mainly from clinical years (62.5%) and the majority were struggling females (62.5%) (Table-I). Our study has explored the experiences of struggling mentees with a formal mentoring program implemented at a private medical college in Rawalpindi. The mentoring program with its fortnightly formal mentoring sessions is responsible to manage the problems of students and to provide psychologist for counselling sessions within the institute. A total of four themes along with twelve subthemes emerged from the data (Table-II).

**Table-I:** Characteristics of the study participants (N=16).

Characteristics		Frequency	Percentage (%)
Gender	Male	6	37.5
	Female	10	62.5
Age	20-22	10	62.5
(Years)	23-25	6	37.5
Year of study (MBBS)	1^st^ Year	0	0
	2nd Year	3 (1M, 2F) 3(F)	18.75 18.75
	3^rd^ Year	5 (3M, 2F)	31.25
	4^th^Year	5 (2M,3F)	31.25
	5^th^ Year		

*M-Male, F-Female.

**Table-II:** Experiences of struggling undergraduate medical students (mentees) with formal mentoring program

Subthemes	Participant’s Quotations
***Theme-1: Psychosocial outcomes of mentoring program***	
Strong mentoring relationship	“I went to my mentor to discuss my problem as she is a very good listener and easily approachable.” (F, Y5, R# 8) “Mentoring depends upon personality of mentor. My mentor keeps smile on his face all the time.” (M, Y5, R# 16)
Emotional, moral, and psychological support	“Formal mentoring program is very helpful in a way that mentors provide continuous support not just once.” (F, Y5, R# 1) “My mother is suffering from cancer. Upon my friend’s suggestion, I discussed with my mentor how to manage time for study and get out of this emotional crisis. My mentor has arranged counselling sessions for me, and I am taking these sessions and improving.” (F, Y2, R# 3) “Mentors use to help students without judging and embarrassing them. I am getting full moral support from the mentoring program. I have found the head of mentoring program to be just like my mother. She often sends me motivational videos and messages.” (F, Y4, R# 9)
Personal & professional development	“My mentor asked me to balance life and career both and consider marriage in my personal development plans. I am planning to take classes for IELT’s in summer vacations so that I can pursue my career abroad after getting married.” (F, Y5, R# 8)
***Theme-2: Mentor as a guide on the side***	
Guidance on research and case-based learning	“My mentor arranged my meeting with the head of scientific society who guided me on various research methods. I am now thinking what area to be chosen.” (F, Y4, R# 6) “Mentors asks us to do patient related study which means whatever cases we see in our clinical rotations; we need to study those topics when we go back home.” (M, Y4, R# 14)
Guidance related to Islam	“Besides studies, mentors guide us about the world and ask us to practice deen and offer prayers.” (M, Y4, R# 14) “In mentoring discussions more knowledgeable mentors omit our ambiguities with reference to Hadith and Quran and guide us the right way.” (M, Y2, R# 15)
Learning from mentor’s life experiences	“Mentors tell their own daily life experiences which are very helpful for us.” (M, Y4, R# 13)
***Theme 3: Mentor as a solution provider to the mentee’s problems***	
Arrangement of extra classes and combined study	“My mentor said that she will arrange combine study for me at the college after college timings. In that case I would be able to study before going home.” (F, Y3, R# 2) “My mentor arranged extra classes for me and few other students as we were facing difficulty in understanding neuroanatomy.” (M, Y2, R# 15)
Helpful mentoring program	“Mentoring program is solving problems of the students that distract them from studies.” (F, Y5, R# 7) “He is helping me on every step. My mentor asks us to do an evening walk or an exercise for stress releasing.” (M, Y4, R# 14)
***Theme 4: Need for structured mentoring program***	
Recruitment of trained and committed staff	“I have felt the need for committed and trained staff for the mentoring purpose. There should be a structured team knowing its job description. That team should be expanded with the passage of time. There should be proper recruitment system to get eligible staff on merit. Mentors should be trained to help mentees according to their issues. They should also do follow up of their mentees.” (F, Y3, R# 5)
Need for verbal feedback from mentees about mentors	“Verbal feedback from the students about mentors is necessary for successful mentoring.” (F, Y3, R# 5)
Need for career counselling	“Main focus of mentoring should be on aspects such as career counselling. Students should get guidance from the start from where they should study for the Part-1 exam. They should be guided about opportunities within the country or abroad and about the various fields of specialization. Mentors only ask us what you are going to do in future they do not actually guide us in choosing our careers.” (M, Y4, R# 11) “There should be numerous sessions for career counseling either within the mentoring program or separately.” (M, Y5, R# 12)
Need for one-to-one mentoring	“There is a need of be one-to-one mentoring because some students cannot share their problems within the group.” (M, Y5, R# 12) “In this program students are unable to share personal things openly. Like you are interviewing us and calling one student at a time rather to talk to all at one time.” (M, Y5, R# 16)

*M-Male, Y-Year of study, R-Respondent

## DISCUSSION

The study has highlighted the experiences of mentees with a formal mentoring program and the useful suggestions provided by them for its betterment. A good mentoring relationship requires some expectations of the mentees from mentors to be fulfilled such as the mentor should be able to make friends and provide personalized guidance.^6^ This relationship later helps in the career development of mentees.^12^ In this study, mentees were satisfied with their mentors’ personal qualities and emotional support. The desired characteristics of a mentor in one of the studies were mutual trust and respect.^13^ But this element of mutual trust and respect is not highlighted in the present study. Mentor should arrange regular meetings with mentees to develop an element of trust.^14^

The mentoring program of the study institute provided emotional support by arranging counselling sessions for a few emotionally drained mentees. Few strategies such as creating positive learning environment, identifying, and assisting struggling students, teaching stress management skills, promoting self-awareness and helping students to promote their personal health can be utilized to promote the well-being of medical students.^15^ Well-being means the absence of distress and the presence of students ‘physical, mental, emotional and spiritual health.^15^ The head of the mentoring program used to motivate one of the struggling mentees by sending motivational videos and messages. One of the past studies showed that mentors used to motivate mentees for better achievements in academics.^16^ Medical students pass through various phases, and they need moral or psychological support to achieve their goals. Mentees in the mentor program of the University of Wisconsin Medical School were psychologically supported.^17^ In another study, mentor roles were to help mentees realize their strengths and minimize weaknesses.^13^ In the present study, mentors provided personal and professional development tips such as time management, self-control and work- life balance to the mentees. In a student support program development of a whole person is focused on rather than just focusing on academics and clinical competence.^18^ Students should be asked to balance their work and life to promote their personal health. 15

This study has also highlighted the guidance provided to the mentees regarding religion and life along with studies. Mentees were provided guidance related to research methods and case-based learning. Students should be facilitated and involved in research in their early years to become physicians and scientists in future.^19^ They should be engaged in case-based learning as it is a collaborative and team-based approach to studies.^20^ The formal mentoring program at the study site is based on the mission of the medical college which states that knowledgeable medical students and good practicing Muslims need to be produced.

The formal mentoring program was helpful for the mentees as it provided solutions to the mentees’ problems. Extra classes were arranged for one of the mentees who had a problem in understanding neuroanatomy and combined study was arranged for a mentee who was unable to study at home due to distractions and commitments. Mentees of the study site gave useful suggestions such as recruiting trained and committed staff, the need for verbal feedback, career counselling and one-one mentoring sessions for the improvement of the existing program. Formal mentoring programs should be adapted to the local context with the training of faculty.^21^ In one of the previous studies^13^ barriers to mentoring were a lack of knowledge about the program and unclear roles within the program. Content of the mentoring program should be carefully chosen that addresses the concerns of the mentees within the context.^13^ One of the participants felt marriage was a hurdle in her career progression until the mentor guided her and told her to balance life and career. Career counselling is necessary to guide students about all the possible specialties of medical field in depth.^22^ In the present study, few mentees provided suggestions regarding one-to-one mentoring sessions so that they can share their personal problems with ease. Vertical peer mentoring can be utilized so that mentees get more time as compared to faculty mentoring which is time-bound.^23^ Individual mentoring can be used for academically weak students, minorities, or psychosocially maladjusted students.^24^

### Strengths of the study:

The study has highlighted the strengths and weaknesses of the formal mentoring program at the study institute. The program can be improved by utilizing the useful suggestions provided by the mentees.

### Limitations of the study:

The study could be conducted at more than one institute to compare mentee’s experiences.

## CONCLUSIONS

The majority of the mentees were satisfied with the formal mentoring program. Literature is evident regarding the reduction of psychological distress faced by medical students with the help of a mentoring program. Mentoring focuses on the personal and professional development of all medical students. Mentoring is fruitful if specific approaches or strategies are added keeping in mind the problems faced by struggling students. Useful suggestions provided by the mentees would help in a better mentoring program at the study site. Furthermore, other institutes can take advantage and implement student support programs for their benefit.

### Authors Contribution:

**AA** conceived the idea and **UM** supervised thesis.

**AA** and **WS** collected data.

**AA** and **WS** did data analysis and interpretation**.**

**AA, WS and LS** drafted manuscript**.**

**UM** did intellectual review of thesis and manuscript.

All the authors contributed towards manuscript and approved submission version. All the authors are accountable for the integrity of research.
